# Deep learning-based automatic meibomian gland segmentation and morphology assessment in infrared meibography

**DOI:** 10.1038/s41598-021-87314-8

**Published:** 2021-04-07

**Authors:** Md Asif Khan Setu, Jens Horstmann, Stefan Schmidt, Michael E. Stern, Philipp Steven

**Affiliations:** 1 Department of Ophthalmology, Faculty of Medicine, University Hospital Cologne, University of Cologne, 50937 Cologne, Germany; 2 Division of Dry Eye and Ocular GvHD, University Hospital Cologne, 50937 Cologne, Germany; 3 ImmunEyez LLC, Irvine, CA USA; 4 Heidelberg Engineering GmbH, 69115 Heidelberg, Germany

**Keywords:** Diagnostic markers, Eyelid diseases

## Abstract

Meibomian glands (MG) are large sebaceous glands located below the tarsal conjunctiva and the abnormalities of these glands cause Meibomian gland dysfunction (MGD) which is responsible for evaporative dry eye disease (DED). Accurate MG segmentation is a key prerequisite for automated imaging based MGD related DED diagnosis. However, Automatic MG segmentation in infrared meibography is a challenging task due to image artifacts. A deep learning-based MG segmentation has been proposed which directly learns MG features from the training image dataset without any image pre-processing. The model is trained and evaluated using 728 anonymized clinical meibography images. Additionally, automatic MG morphometric parameters, gland number, length, width, and tortuosity assessment were proposed. The average precision, recall, and F1 score were achieved 83%, 81%, and 84% respectively on the testing dataset with AUC value of 0.96 based on ROC curve and dice coefficient of 84%. Single image segmentation and morphometric parameter evaluation took on average 1.33 s. To the best of our knowledge, this is the first time that a validated deep learning-based approach is applied in MG segmentation and evaluation for both upper and lower eyelids.

## Introduction

Dry eye disease (DED) is one of the pervasive diseases of the ocular surface due to its multifactorial nature where tear film instability, hyperosmolarity, neurosensory abnormalities, ocular surface inflammation, ocular surface damage, and meibomian gland dysfunction (MGD) play etiological roles^1–4^. DED affects visual acuity and causes ocular discomfort and other symptoms, leading to changes in the quality of life^2,5^.

MGD is a chronic disease and caused by diffuse abnormalities of meibomian glands, terminal duct obstruction, and changes in the glandular secretion. Obstructive MGD is a common cause of evaporative dry eye and lipid layer deficiency^5–7^. Infrared (IR) Meibography is a well-established non-contact optical imaging technique, which uses IR illumination to depict MG morphology by examining the everted eyelid^7^. Clinically, it is widely accepted, and also recommended to image and quantify MG during dry-eye examination. Meibography is non-invasive, provides large image areas, detailed morphometric information of MG, and is easy to operate for clinicians and technicians.

Several grading schemes and methods to analyze MG have been published by Pult et al.^8^, Srinivasan et al.^9^, Arita et al.^10^, and Engel et al.^11^ However, these MG segmentation methods are subjective or semi-automatic and require user interaction, which is laborious, time-consuming and non-repeatable. In previous research^8–11^, meibography images have been segmented and quantified using ImageJ (National Institute of Health; http://imagej.nih.gov/ij) software where clinicians need to involve manually to identify the MG. In addition, different clinicians identify glands differently, which causes inter-observer variability^12^.

A reliable automatic MG segmentation technique may overcome the difficulties of manual image segmentation, as infrared meibography images often contain various artifacts such as low contrast, non-uniform illumination, defocus gland area, or specular reflections which make image segmentation more challenging^13^. Until now, Koh et al.^12^, Llorens-Quintana et al.^13^, Arita et al.^14^, Celik et al.^15^, and Koprowski et al.^16^ have proposed automatic MG segmentation methods. However, all of these methods rely on intensity-thresholding based image segmentation which performance heavily depends on image quality. At the same time, it is quite challenging for examiners to repeatedly acquire sufficient quality images.

In contrast to intensity-thresholding based image segmentation methods, a deep learning-based method has the advantages of learning useful features and representations from the raw images automatically^17^ and could thus overcome the above-mentioned restrictions. In a previous study^18^, we have compared intensity-thresholding, region growing, and deep learning method to automatic analysis of IR meibography images. Among these three implemented approaches, we have demonstrated that deep learning could produce high quality and reliable results for the challenging task of automated IR meibography image segmentation and quantification. In the past years, deep learning has been paid increasing attention in the field of ophthalmology^19–22^. Using a neural network and a high volume of image data, a deep learning algorithm can learn to detect specific objects from the images. Nowadays, deep learning algorithms perform well due to increased computational power and image data. U-net, a state-of-the-art biomedical image segmentation method was first introduced by Ronneberger et al.^23^ The algorithm provides high performance while requiring less training image data and gaining more accuracy than the conventional neural network.

In this research, an automatic MG segmentation method is proposed based on U-net. The detailed deep learning model training procedure and application to new images is illustrated in supplementary Fig. S1.

Unlike previous image segmentation techniques whose performance heavily depends on the image quality, the proposed deep learning-based MG segmentation method directly learns the MG features from the training images, which does not require any pre-processing such as artifact removal or image enhancement. It can automatically segment MG on new images. We demonstrate the good performance of the proposed technique by assessing various evaluation metrics.

## Materials and methods

### Meibography data collection

In total 728 anonymized clinical infrared meibography images of both upper (398) and lower (330) eyelids of adults male and female humans (age > 18 years) were randomly collected using the database of an Oculus Keratograph 5 M (Oculus GmbH, Wetzlar, Germany) device at the Eye Hospital, Department of Ophthalmology, University Hospital Cologne, Germany. The image collection adhered to the tenets of the Declaration of Helsinki and was approved by the Ethics and Institutional review board (IRB) from the University of Cologne—Germany (approval number #16-045). As this research study was conducted retrospectively using anonymized non-biometric data, the need for participant’s informed consent was waived by the Ethics committee and IRB.

### Data annotations

**Figure 1:**
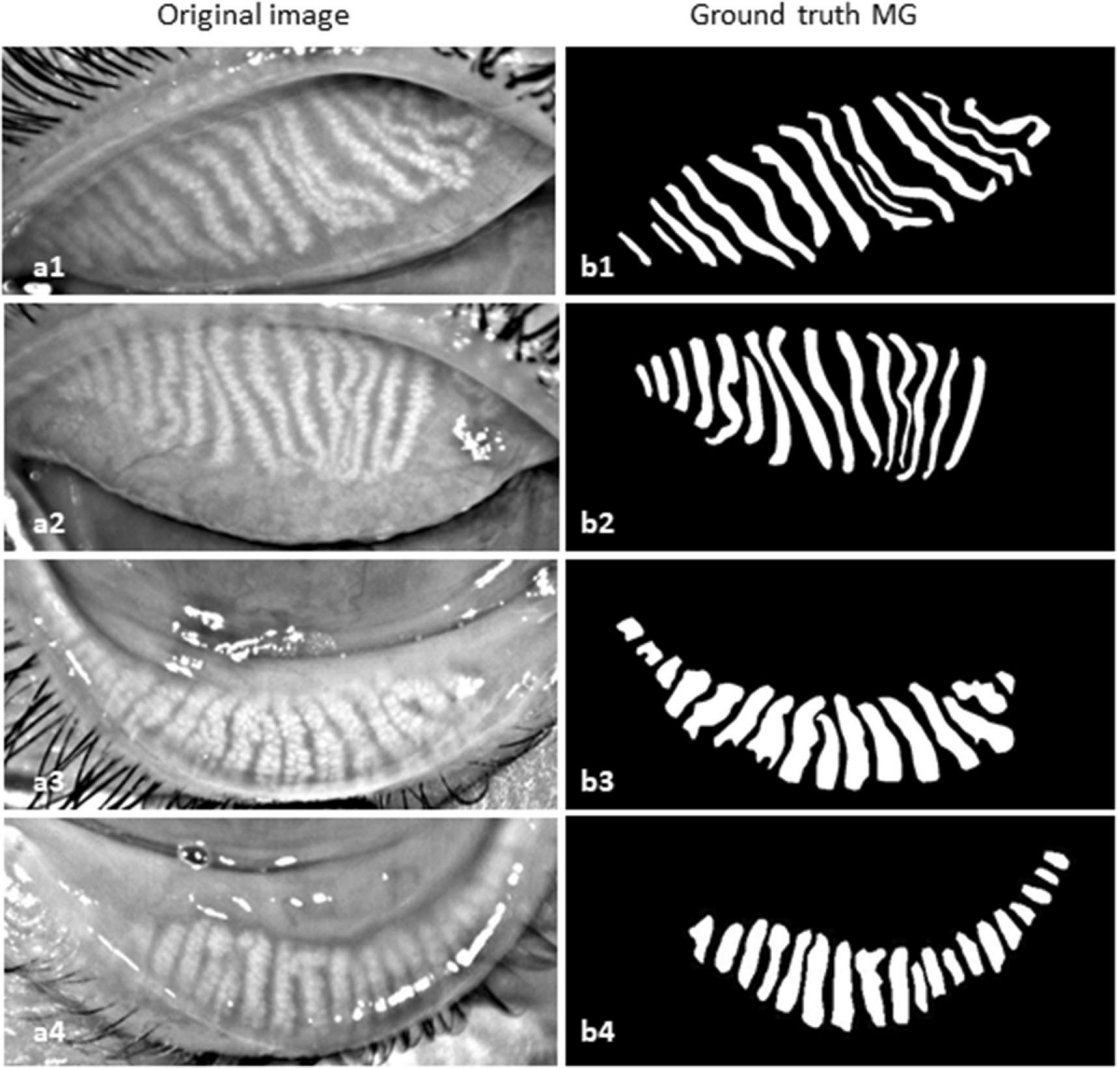
Example of training images and their corresponding ground truth images. (**a1–a2**) anonymized upper eyelid images, (**a3–a4**) anonymized lower eyelid images, (**b1–b2**) upper eyelid ground truth images, and (**b3–b4**) lower eyelid ground truth images.

All collected clinical meibography images were manually annotated using Fiji (fiji.sc), which is an open-source image processing software, with the Segmentation Editor (https://imagej.net/Segmentation_Editor) plugin to generate ground truth masks. In the segmentation editor plugin window, the Exterior was set to black, and the Interior was set to white to generate a binary ground truth mask. One of the authors (M.A.K.S.) created a polygon boundary around the glands and saved the annotation mask images as JPG file format. Ground truth masks were generated for both upper and lower eyelids. Examples of training images and their corresponding masks of upper and lower eyelids are presented in Fig. 1. Finally, all annotated ground truth mask images were verified and corrected by an experienced senior ophthalmologist (P.S.) from the University Hospital Cologne before they were used in deep learning model training and testing.

### Data preparation and allocation

From the overall collected 728 anonymized clinical meibography images, 100 were prepared as a test dataset to measure the true performance of our trained model. The remaining 628 images were randomly divided into training and validation datasets. Approximately, 80% (502 images) and 20% (126 images) of image data were used to train and validate respectively the deep learning model. Due to variations in the size of data set images, all images were resized to 256 × 256 pixels (width × height). Training images were used for deep learning model training and validation images were used to fine-tune the hyper-parameters such as epochs, learning rate, momentum, validation steps.

### Deep learning model design and training

We have applied transfer learning with a pre-trained backbone to increase the learning efficiency of the deep learning model. In this research work, Inception-ResNet-v2^24^, a pre-trained backbone based on a publicly available ImageNet^25^ dataset was used for transfer learning. In the beginning, we pre-trained our model with the Montgomery Chest X-ray images^26^ for five epochs, which helps transfer learning performance as such medical image data is closer to our target application than the original ImageNet dataset. There are 138 publicly available X-ray images acquired by the Department of Health and Human Services of Montgomery County, MD, USA. The approach is to pre-train the baseline U-Net model to increase learning efficiency. After that, we re-trained the U-net model again using the meibography images and their respective masks. To optimize the deep learning model, both cross-entropy (CE) and Dice loss were used. The CE loss, Dice loss, and total loss were computed by the following equations,

1 $${\text{CE}} = \mathop \sum \limits_{x \in \varOmega } w\left( x \right)g_{l} \left( x \right)\log \left( {p_{l} \left( x \right)} \right)$$

2 $$Dice = 1 - \frac{{2 \Sigma_{x \in \Omega } p_{l} \left( {\text{x}} \right)g_{l} \left( {\text{x}} \right)}}{{\Sigma_{x \in \Omega } g_{l}^{2} \left( {\text{x}} \right) + \Sigma_{x \in \Omega } p_{l}^{2} \left( {\text{x}} \right)}}$$

3 $$Total\;loss = CE + Dice$$

where w(x) is the weight assigned to the pixel $x \in \varOmega$, $g_{l} \left( {\text{x}} \right)$ is the ground truth pixel for layer $l$, $p_{l} \left( x \right)$ is the segmented pixel for layer $l$ and total loss is the summation of the cross-entropy and dice loss.

Adam^27^, a gradient-based stochastic optimizer that is one of the most efficient optimization algorithms for deep learning model optimization was also used to optimize the deep learning model.

### Cross-validation study

To evaluate a deep learning model k-Fold cross-validation is generally used. In this research, k = 5 was applied. The total 628 images were randomly split into 5 sub-groups and each time four groups were selected as a training dataset and one group was selected as a validation dataset. In total 502 and 126 images were used as training and validation datasets respectively on every fold for training the model.

### Evaluation metrics

For the MG segmentation task in this research, the common evaluation metrics Precision (P), Recall (R), and F1 score have been used. The MG pixels (e.g. white pixels in the binary segmented masks) are considered as positive instances. Based on the combination of the ground truth masks and segmented masks, these pixels are categorized into four categories: true positive (TP), true negative (TN), false positive (FP), and false negative (FN). Then we can interpret Precision, and Recall in the following equations:

4 $$P = \frac{TP}{{TP + FP}}$$

5 $$R = \frac{TP}{{TP + FN}}$$

F1 score is interpreted as a weighted average of precision and recall and based on the following equation:

6 $$F1 = \frac{2 \cdot P \cdot R}{{P + R}}$$

### Morphometric parameters assessment

To better analyze the MG’s morphology, gland number, length, width, and tortuosity were measured. These morphometric parameters were computed directly from the output of a deep learning-based binary segmentation image. To calculate the morphometric parameters, each segmented gland was analyzed individually. However, to generalize these morphometric parameters, the mean gland length, width, and tortuosity were calculated for an individual eyelid.

**Figure 2:**
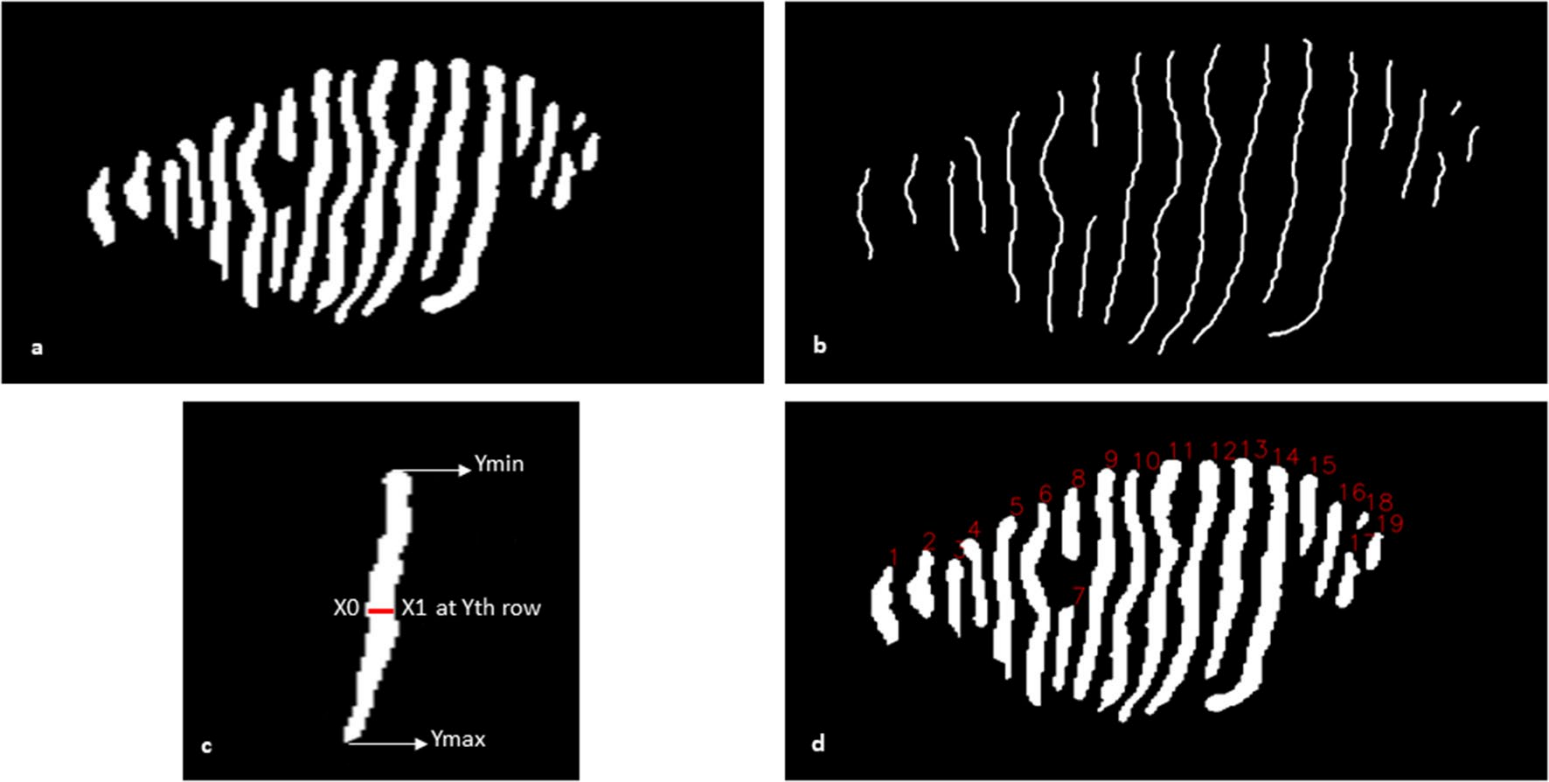
Morphometric parameters assessment of MG. (**a**) Binary segmented image, (**b**) skeletonized MGs of one-pixel width, (**c**) Individual gland width measurement. The Red line indicates the leftmost white pixel (X_0_) and rightmost white pixel (X_1_) at the Y_th_ row, and (**d**) Number of each MG is presented on top of each gland.

To compute the individual gland width, the difference of the leftmost white pixel (X_0_) and the rightmost white pixel (X_1_) at each row (Y) within the range of the length of the gland (Y_min_, Y_max_) were measured and then averaged (Fig. 2c). The number of MGs were computed based on the total number of separated white objects in the binary segmented image and put the number on top of each MG (Fig. 2d).

To compute the length and tortuosity of the gland, first, the binary-segmented image (Fig. 2a) was skeletonized (Fig. 2b) into one-pixel width using a custom-made python-based software. Then the path length (distance over the gland’s skeletonized path) and the chord length (Euclidean distance between the first and last pixel of an individual skeletonized gland) were calculated. Finally, tortuosity $\tau$ for each gland g was measured as:

7 $$\tau \left( g \right) = \frac{Path\; length}{{Chord\; length}}$$

The deep learning model training, validation, testing, data preparation, and morphometric parameters assessment were conducted on a laptop running on Windows 10 Professional, 64-bit Intel Core i7-9750H CPU @ 2.6 GHz with 12 MB of cache memory, SSD 512 GB M.2 Samsung 970 Pro PCIe 3.0 × 4 NVMe, RAM 32 GB DDR4 @ 2666 MHz and NVIDIA GeForce RTX 2070 Max-Q with 8 GB GDDR6 of memory. Data preparation, deep learning model design, and training, morphometric parameters assessment software was written in Python (version: 3.6.6) using Keras (version: 2.2.5) with TensorFlow (TensorFlow version: 1.4, CUDA version: 10.0, cuDNN version: 7.6.3) in the backend.

### Test–retest reliability

To determine the variability and repeatability (test–retest reliability)^28,29^ of the developed algorithm, we additionally have compared two manual annotations by the same observer with two automatic segmentations, using the same test dataset of 100 images (50 upper and 50 lower eyelids). The same observer (M.A.K.S.) manually re-annotated individual MG of the test dataset. Dice similarity coefficient (DSC)^30^, Cohen’s kappa coefficient^31^, and inter-class correlation coefficient (ICC)^32^ were used to measure the variability and repeatability between manual annotations and between automatic segmentations of the test dataset.

### Statistics

The deep learning-based MG segmentation method was compared with the manually segmented MGs. The performance of the deep learning-based segmentation was measured using the Bland–Altman method^33^. Open-source program Python (version 3.6.6) based SciPy (version 1.5.2) library was used to perform the statistical analysis.

## Results

### Meibomian glands segmentation

**Figure 3:**
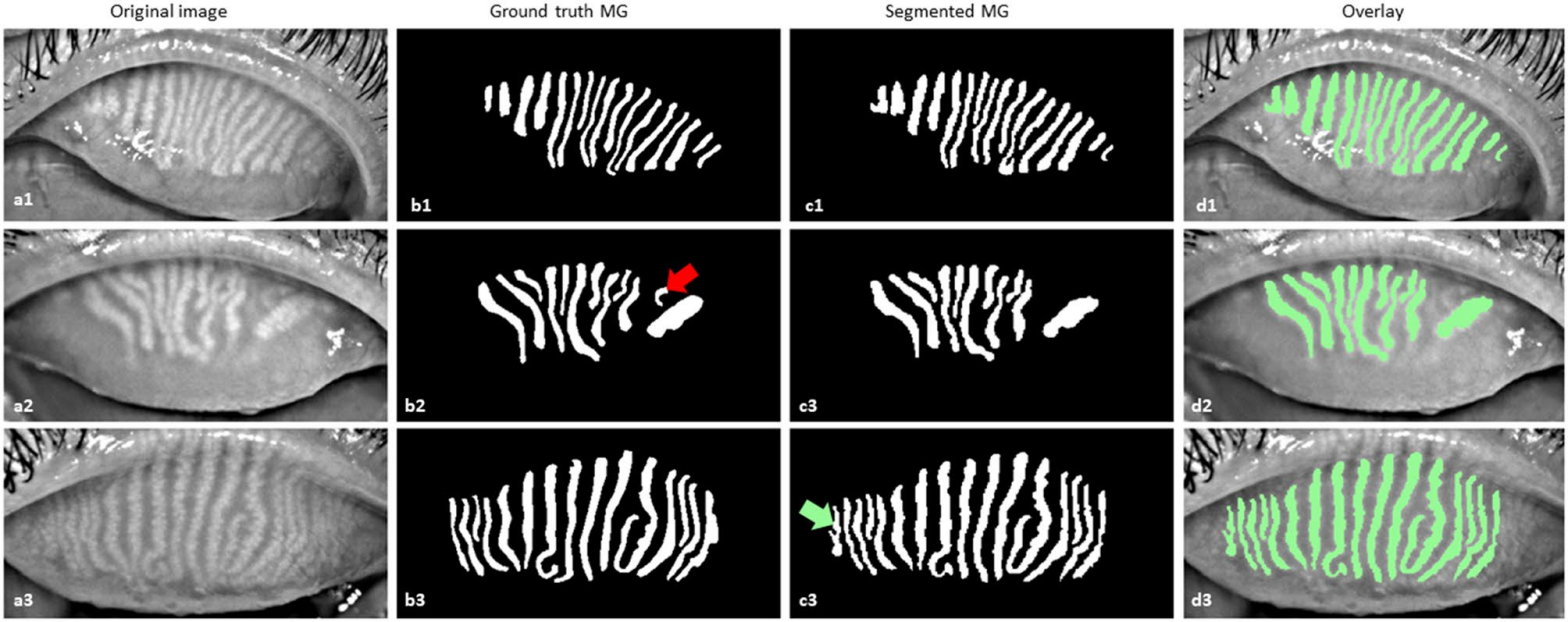
Example of MG segmentation from upper eyelid on test images. (**a1**–**a3**) Original images, (**b1**–**b3**) ground truth MG, (**c1**–**c3**) segmented MG, and (**d1**–**d3**) overlay of the original image and segmented MG. The red (**b2**) and green (**c3**) arrows indicate the missing gland and false gland segmentation respectively.

**Figure 4:**
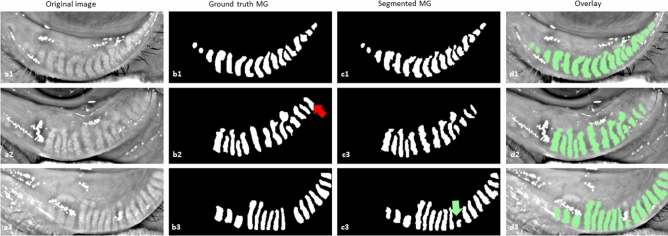
Example of MG segmentation from lower eyelid on test images. (**a1**–**a3**) original images, (**b1**–**b3**) ground truth MG, (**c1**–**c3**) Segmented MG, and (**d1**–**d3**) overlay of the original image and segmented MG. The red (**b2**) and green (**c3**) arrows indicate the missing gland and false gland segmentation respectively.

MG segmentation model training for 30 epochs with a batch size of 5 took approximately 8 h and 24 min using the above described hard- and software. To segment and evaluate all 100 testing images, took 1 min 58 s on average 1.33 s per image. The average precision, recall, and F1 score were achieved 83%, 81%, and 84% respectively on the testing dataset with an AUC value of 0.96 based on the ROC curve (Supplementary Fig. S2) and dice coefficient of 84%. Examples of upper and lower eyelid testing images segmentation are presented in Figs. 3 and 4.

**Figure 5:**
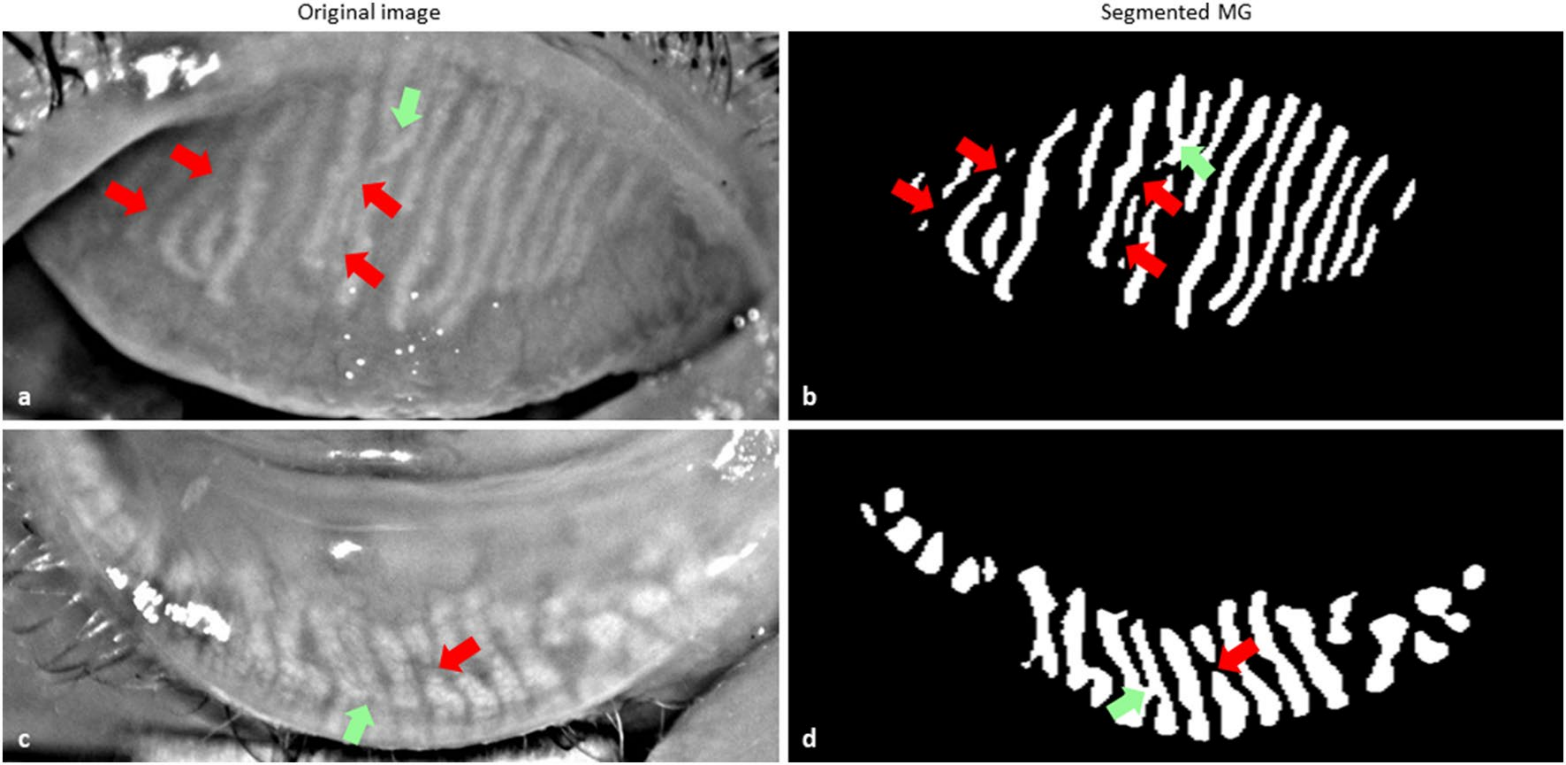
Example of MG segmentation where MG did not segment accurately. (**a**,**c**) Original images. (**b**,**d**) Segmented MGs. Green arrows indicate the MGs where glands need to appear separate and red arrows indicate where MGs need to become continuous.

In general, MGs were reliably segmented in all testing images of both upper and lower eyelids using the trained deep learning model. However, the glands were not accurately segmented in all-test images. In some cases, two glands were connected which need to appear separately and the single gland was divided into two, which need to be a continuous gland. Examples of segmented MGs where glands are connected and separated are illustrated in Fig. 5. Among the 100 upper (50) and lower (50) eyelid test images, 39 upper and 37 lower eyelid images were segmented correctly. One MG was missing in 6 upper and 7 lower eyelid images while two MGs missing in only one lower eyelid image. Furthermore, one MG was falsely segmented in 4 upper and 5 lower eyelid images while two MGs falsely segmented only one upper eyelid image.

### Morphometric parameters assessment

The morphometric parameters MGs number, length, width, and tortuosity were computed from the binary-segmented images of test dataset. These are important clinical parameters to analysis the MG status. Our developed Python-based software provides automatic morphometric parameters assessment from the binary segmented image. All morphometric parameters of the binary segmented images of test dataset for both upper and lower eyelids were compared with the manually annotated images and the *p* value (using paired t-test) between ground truth and automatic segmentation is also presented. The results are tabulated in Tables 1 and 2.

**Table 1:** Morphometric parameters analysis of upper eyelid.

Upper eyelid parameters	Ground truth MG	Segmented MG	*p* value
Number of glands	15.02 ± 2.68	15.00 ± 2.61	0.79
Mean gland length	269.87 ± 188.78	249.20 ± 181.65	0.58
Mean gland width	18.14 ± 2.95	19.20 ± 2.81	0.07
Mean tortuosity	1.74 ± 0.95	1.68 ± 0.91	0.18

*p* value is between ground truth versus segmented MGs.

**Table 2:** Morphometric parameters analysis of lower eyelid.

Lower eyelid parameters	Ground truth MG	Segmented MG	*p* value
Number of glands	15.41 ± 3.18	15.32 ± 3.19	0.32
Mean gland length	105.60 ± 90.21	99.96 ± 87.36	0.09
Mean gland width	22.33 ± 5.40	22.66 ± 5.21	0.04
Mean tortuosity	1.78 ± 0.94	1.83 ± 0.94	0.61

*p* value is between ground truth versus segmented MGs.

**Figure 6:**
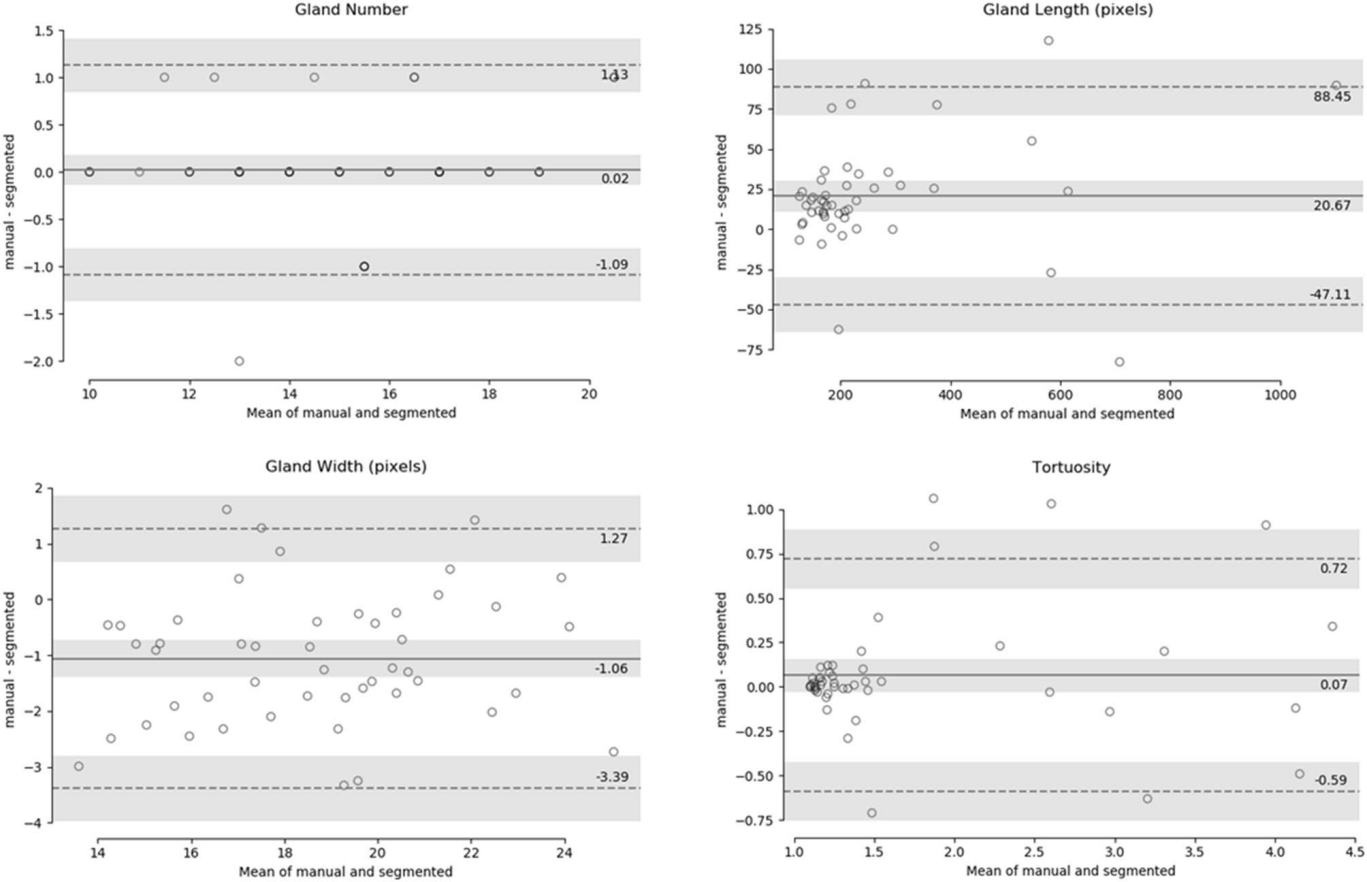
Bland–Altman plots of MGs number, length, width, and tortuosity between ground truth and deep learning segmentation for upper eyelid. The middle solid line indicates the mean value, the two dotted lines indicate the limit of agreement (± 1.96 SD), and the gray bar indicates the confidence interval of 95%.

To determine the consistency of the automatic MGs segmentation and manual annotation, Bland–Altman analysis was performed for all morphometric parameters. The statistical plots for upper eyelid are presented in Fig. 6. The total 50 upper eyelid testing images were used for this analysis. The middle solid line indicates the mean of manual and segmented MG which are 0.02, 20.67, − 1.06 and 0.07 for gland number, length, width and tortuosity respectively. All mean values are close to zero, thus the ground truth and automatic segmentation are not significantly different (*p* > 0.005). The two dotted lines indicate the limit of agreement (+ 1.96 SD) and the values were in between 1.13 and − 1.09 for gland number, 88.45 and − 47.11 for length, 1.27 and − 3.39 for width and 0.72 and − 0.59 for tortuosity. The gray bar indicates the confidence interval of 95%. Among the all 50 test images only 1, 2, 0, and 3 values were outside the limit of agreement thus the 98%, 96%, 100%, and 94% of the values are within the limit of agreement.

**Figure 7:**
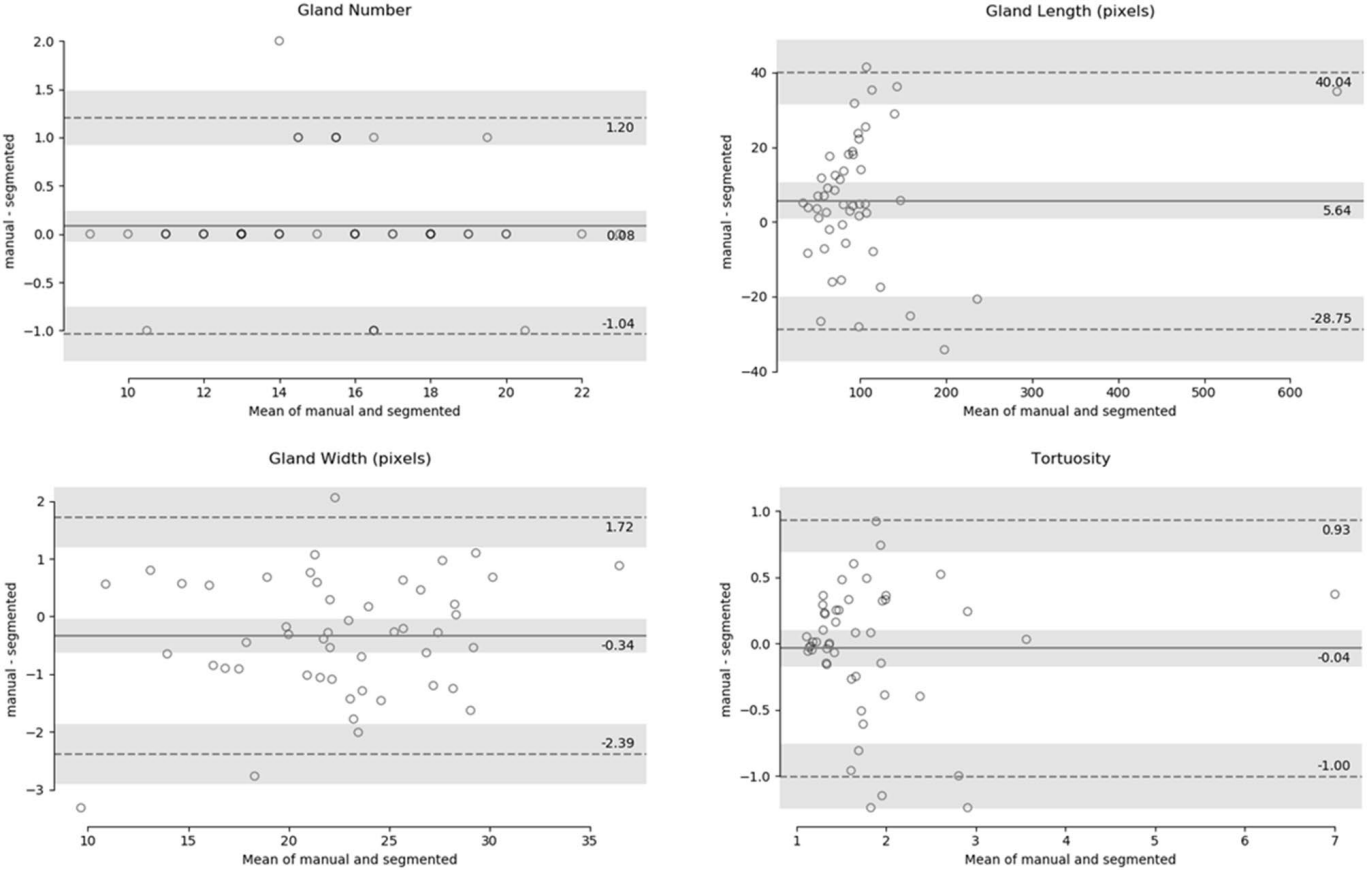
Bland–Altman plots of MGs number, length, width, and tortuosity between ground truth and deep learning segmentation for lower eyelid. The middle solid line indicates the mean value, the two dotted lines indicate the limit of agreement (± 1.96 SD), and the gray bar indicates the confidence interval of 95%.

Figure 7 represents the lower eyelid analysis. The total 50 lower eyelid testing images were used for this analysis. The middle solid line indicates the mean of manual and segmented MG which are 0.08, 5.64, − 0.34 and − 0.04 for gland number, length, width and tortuosity respectively. All mean values are close to zero, thus the ground truth and automatic segmentation are not significantly different (*p* > 0.005). The two dotted lines indicate the limit of agreement (± 1.96 SD) and the values were in between 1.20 and − 1.04 for gland number, 40.04 and − 28.75 for length, 1.72 and − 2.39 for width, and 0.93 and − 1.00 for tortuosity. The gray bar indicates the confidence interval of 95%. Among the all 50 test images only 1, 0, 1, and 2 values were outside the limit of agreement thus the 98%, 100%, 98%, and 96% of the values are within the limit of agreement.

### Test–retest reliability

The developed algorithm achieved 0% variability and 100% repeatability, while running two tests, in contrast, there was variability between two manual annotations with the same observer and using the same test dataset. The variability and repeatability between manual annotations (intra-observer) and between automatic segmentations (intra-method) are presented in the Table 3.

**Table 3:** Variability and repeatability analysis of intra-observer and intra-method on test dataset images.

Metrics	Upper eyelid		Lower eyelid	
	Intra-observer	Intra-method	Intra-observer	Intra-method
DSC	0.96 ± 0.01	1.0 ± 0.0	0.97 ± 0.01	1.0 ± 0.0
Kappa	0.87 ± 0.04	1.0 ± 0.0	0.83 ± 0.08	1.0 ± 0.0
ICC	0.90 ± 0.06	1.0 ± 0.0	0.86 ± 0.11	1.0 ± 0.0

Intra-observer = between manual annotations of the same observer, Intra-method = between automatic segmentations running two tests.

### Performance evaluation of inferior quality images

**Figure 8:**
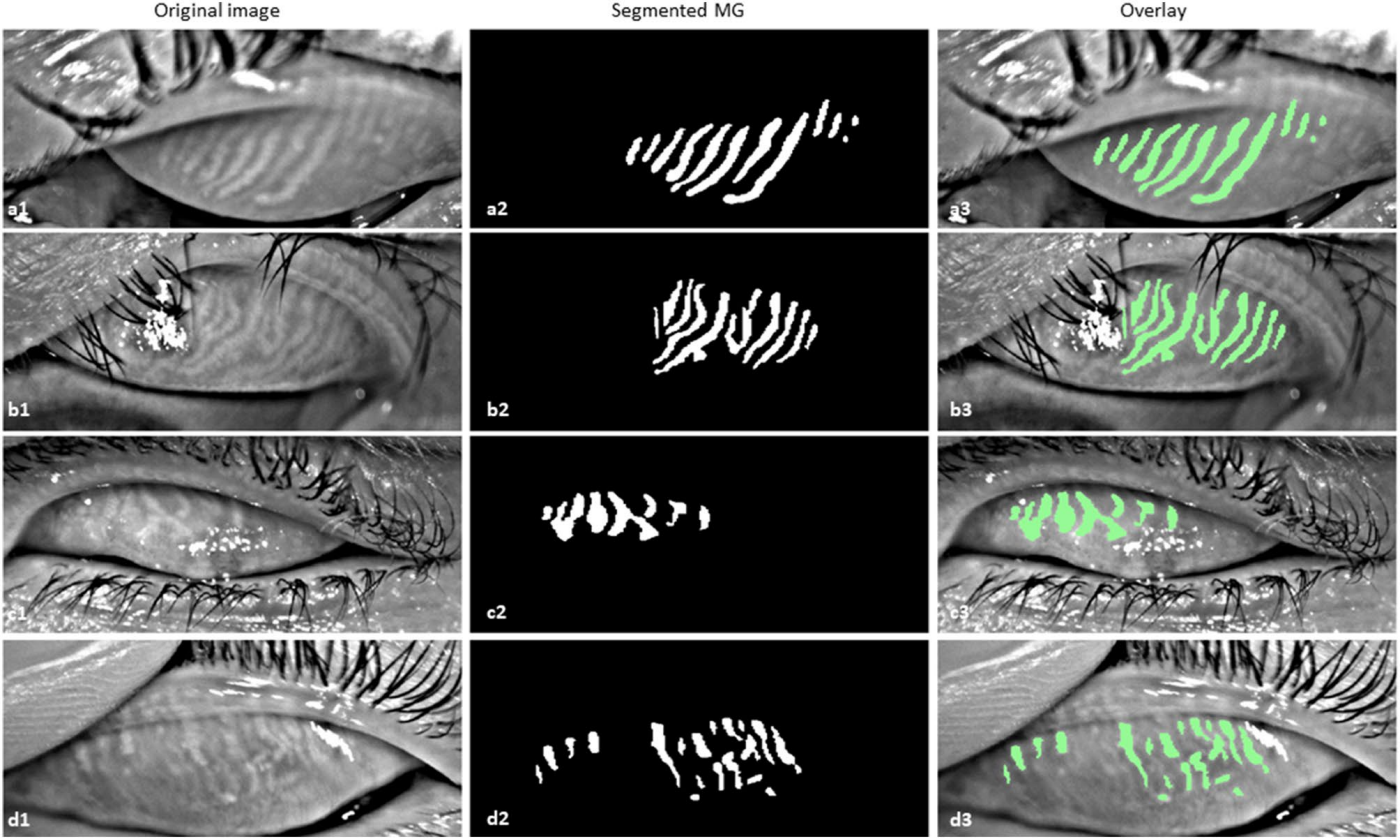
Example of MG segmentation on inferior quality images. (**a1**–**a3**) Out of focus image, segmented MGs, and overlay, (**b1**–**b3**) Eyelashes in the glands area image, segmented MGs, and overlay, (**c1**–**c3**) Partially everted upper eyelid image, segmented MGs, and overlay, and (**d1**–**d3**) Artifact of everting finger in the top left of the image, segmented MGs, and overlay.

The trained deep learning model was also able to segment MGs from different inferior quality of meibography images, which were not considered during the deep learning model training, validation, and testing such as out of focus image, eyelashes in the glands area, partially everted upper eyelid, and artifact of everting finger in the top left of the image. Examples of MGs segmentation on inferior quality images are illustrated in Fig. 8.

## Discussion

In several previous publications, MGs were detected using the image processing software ImageJ^8–11^. User interaction is needed with this software to define the MGs manually for each image to quantify the meibography images. Users may draw the gland regions differently and therefore intra- or inter-observer variability occurs. The first automatic MG detection algorithm was published by Koh et al.^12^ and the algorithm was able to detect glands from both healthy and unhealthy glands. The most important part of this research work depended on how the meibography images were acquired. Small artifacts may cause complications during the MG detection and thus the final result was affected. Later, another automated algorithm for quantitative analysis of MG was developed by Arita et al.^14^ based on image enhancement methods where various image processing techniques were applied. In their research work, user interaction was needed for a manual correction after the MG detection when the images had too many specular reflections and extensive MG loss. Thus, the algorithm was not fully automatic. In recent works, Llorens-Quintana et al.^13^ proposed a fully automatic MG detection based on image enhancement methods using various image processing techniques. In their research work, they also proposed a gland irregularity measurement which was very helpful for clinicians for a follow-up checkup. However, user input was necessary, and the algorithm did not work for several images as they were not taken properly and there were some artifacts such as defocus areas, out of frame image and everted upper eyelid touching the lower eyelid. Additionally, this algorithm was designed only for the upper eyelid MG detection.

A machine learning-based automatic MG detection was developed by Celik et al.^15^, which was able to detect MG using the Gabor filter. A supervised machine learning algorithm, support vector machine (SVM), was used to classify the pixels that belong to either glands or inter-glands areas. However, while our model is trained with a gland class and a general background class, their model solely learns from the gland and inter-gland areas, which could potentially lead to false detection. Furthermore, this algorithm was tested for the upper eyelid. Another artificial intelligence (AI) based MG detection was proposed by Koprowski et al.^16^, where the SVM classifier was used to classify healthy, at-risk, and affected subjects with very high sensitivity (99.3%) and specificity (97.5%). The main limitations of this algorithm were the use of handcrafted features and preprocessing, which provided only the centerline information of each MG and there were no options to separate the glands. Also, the quantification of MGD was not possible using global analysis, and the AI (SVM classifier) sometimes produced false results due to the lack of classification features.

Unlike the traditional AI, deep learning-based methods do not require handcrafted features or image preprocessing and it extracts the useful features from the raw images automatically^17^ using the hidden layers. First‚ deep learning-based MG segmentation and morphometric quantification based on U-Net architecture was proposed by Prabhu et al.^34^ They achieved acceptable *p* values (*p* > 0.005) between manual annotation and automatic segmentation method. However, comparison to our model performance is not possible as no deep learning performance metrics were mentioned. The authors calculated gland width by measuring the pixels exclusively at the mid-line of the automatic segmented image. In our research study, we instead calculated individual gland width by measuring the difference of the leftmost pixel and the rightmost pixel at each row within the range of the length of the gland and then averaged. Furthermore, in contrast to the previously published model, which was trained for 300 epochs without transfer learning, our model was trained using two stage transfer learning, enabling 30 epochs, which is more efficient and computationally inexpensive^35^. In addition, their proposed method tested only upper eyelid meibographies. Another deep learning-based MG segmentation based on pyramid scene parsing (PSP) network has been proposed by Wang et al.^36^, where they achieved 95.4% accuracy and 66.7% intersection over union (IoU) to analyze gland atrophy regions. In contrast, our proposed U-Net based method achieved 84% dice coefficient to segment individual MGs. Maruoka et al.^37^ proposed a deep learning-based method to detect obstructive MGD using in vivo confocal microscopy (IVCM). They achieved 94.2% sensitivity and 82.1% specificity to detect normal and obstructive MG acini. Though IVCM provides high resolution images but it is not routinely used to image meibomian gland, and their field of view is small to analyses the full length of MG or total area of the eyelid.

Our research study proposed a deep learning-based method to automatically segment and evaluate various morphometric features of MGs from IR meibography images without any image pre-processing. This deep learning model achieved high performance with 0% variability and 100% repeatability (test–retest reliability). The average computational time for segmentation and evaluation per IR meibography image was approximately 1.33 s (experiments were performed on above mentioned laptop). To evaluate 1000 disease images our method could take only 22 min without any clinicians’ involvement within a single click which reduces significant processing time and computational cost. Furthermore, statistical analysis presents that more than 95% of all morphometric parameters value for both upper and lower eyelids are within the limit of agreement. Our proposed method achieved very high performance with on average 84% dice coefficient. The single trained model is able to segment MG of both upper and lower eyelids. From the visualization of the automated segmentation of MGs have high visual similarity with manual ground truth MGs. Overall, our research results demonstrate a fully automated and reliable deep learning-based technique for MG segmentation and morphometric evaluation from IR meibography images.

This research work has some limitations. Firstly, the proposed deep learning-based technique is device-specific, where training, validation, and testing images were acquired using a Keratography 5 M device. However, for the training of the neural network, MG features were automatically selected from training images by the model itself. Thus, the algorithm may segment MG from images of other IR meibography devices that need to be tested and validated. Secondly, all ground truth masks were generated manually where there is a possibility of inter-observer variability. However, all ground truth masks were validated and corrected by an experienced clinician. Finally, MGs are not accurately segmented in all test images. In some cases, two glands are connected, which need to appear separately and in other cases, single glands are separated into two or more glands, which need to be a continuous gland (Fig. 5). In all of these cases, meibography images were not acquired correctly and the acquisition problems were encountered due to the strong specular reflections, unfocused image, and MGs morphology. From Fig. 5a and c, it is clearly visible in the original images, where the glands are separated (red arrows), and the glands are connected (green arrows), that the images were not acquired correctly and the MGs are connected or overlapping. To overcome these limitations, in the future, we plan to increase the number of training dataset to more than 1000 patients and increase the number of inferior quality images thus the trained model could learn the MG features more precisely. This would also increase the learning efficiency of the deep learning model and able to segment MG more accurately from the inferior quality of images.

## Conclusions

A deep learning-based automated IR meibography image segmentation method has been proposed in this research work. It reliably segments the MG as well as provides automated morphometric evaluation. The proposed technique overcomes the limitations of subjective MG segmentation and assessments which enables faster, non-invasive, precise, accurate and reproducible MG characterization thus specify better and faster disease diagnosis, new drug development, and clinical trials for a new drug of MGD related DED. These automated analyses provide valuable objective information regarding MG, which reduces, inter- or intra-observer variability and time associated with manual perception to analyze the large volume of clinical images.

## Supplementary Information

Supplementary Figures.

## Data Availability

The image data utilized in this study are not publicly available due to the patients’ privacy.
