# Supplementary material for: Deep learning-based automatic meibomian gland segmentation and morphology assessment in infrared meibography
Source: Sci Rep. 2021 Apr 7;11:7649. doi: 10.1038/s41598-021-87314-8 (PMC8027879; doi:10.1038/s41598-021-87314-8)
Supplement: Supplementary file 1 — Supplementary Figures. [file 41598_2021_87314_MOESM1_ESM.docx]

Deep Learning-based Automatic Meibomian Gland Segmentation and Morphology Assessment in Infrared Meibography

Md Asif Khan Setu^1,2^, Jens Horstmann^1^, Stefan Schmidt^4^, Michael E. Stern^1,2,3^, Philipp Steven^1,2,*^

^1^ Department of Ophthalmology, University of Cologne, Faculty of Medicine and University Hospital Cologne, Cologne, 50937, Germany

^2^ Division of Dry Eye and Ocular GvHD, University Hospital Cologne, Cologne, 50937, Germany

^3^ ImmunEyez LLC, Irvine, CA, USA

4 Heidelberg Engineering GmbH, Heidelberg, 69115, Germany

*Correspondence: [philipp.steven@uk-koeln.de](mailto:philipp.steven@uk-koeln.de)


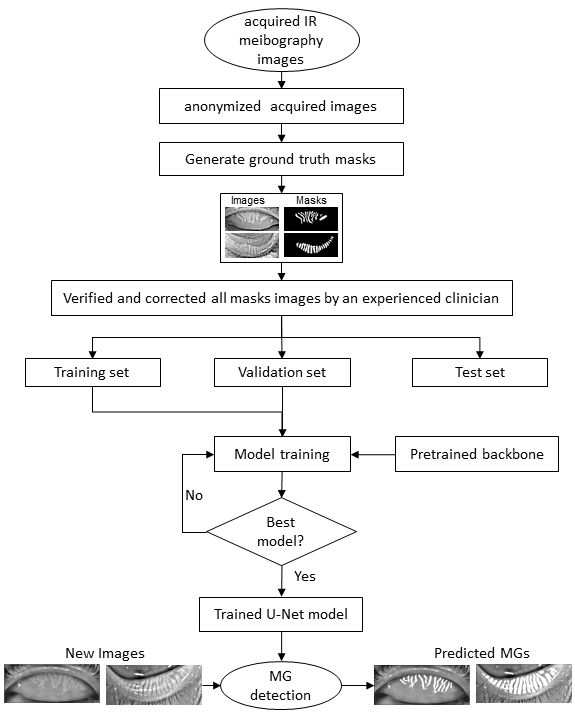


**Figure S1.** Deep learning model training procedure and application to new images.


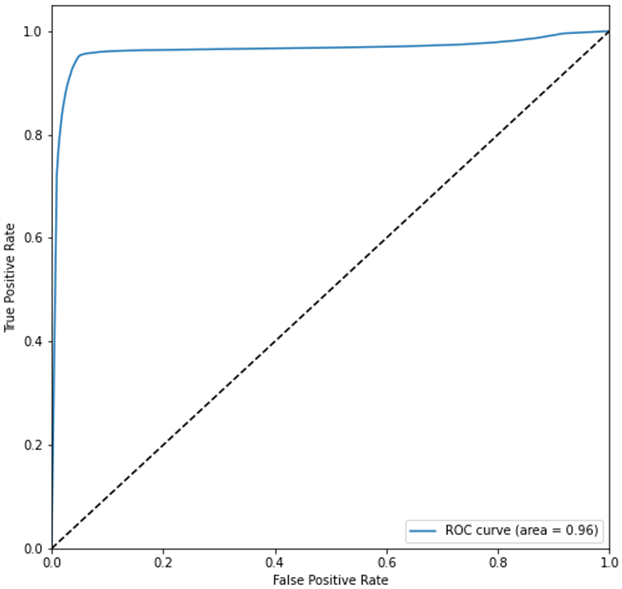


**Figure S2.** Receiver operating characteristic (ROC) curve between ground truth and deep learning segmentation. The AUC value of the deep learning model is 0.96 based on ROC curve.
